# The complete chloroplast genome of ‘black tiger 2’ (*Kadsura coccinea* (lem.) A.C. Smith) in southeast of China and phylogenetic relationshipsAQ1

**DOI:** 10.1080/23802359.2019.1698328

**Published:** 2019-12-13

**Authors:** Jun Yang, Xiao-An Wang, Ming-Yao Gao, Xiao-Xia Wei

**Affiliations:** Fruit Research Institute, Fujian Academy of Agricultural Sciences, Fuzhou, China

**Keywords:** *Kadsura coccinea* (Lem.) A.C. Smith, black tiger 2, complete chloroplast genome, phylogenetic analysis

## Abstract

A new super *Kadsura coccinea*, named ‘black tiger 2’, was selected from variant forms of seedlings. In this study, The complete chloroplast (cp) genome of ‘black tiger 2’ was obtained. The complete cp genome is 145,608 in length, and contained 126 genes, including 83 protein-coding genes, 8 ribosomal RNA genes, and 35 transfer RNA genes. Phylogenetic analyses established that ‘black tiger 2’ was closely clustered with other Schisandraceae species such as *Schisandra chinensis* and *Illicium*, which helps elucidate the phylogenetic relationship between ‘black tiger 2’ and other species.

*Kadsura coccinea*, also known as ‘black tiger’ in China, is a woody vine plant originating from south China, belonging to the Schisandraceae family (Sun et al. 2009). It is mainly distributed in Fujian, Guangxi, Guizhou, Yunnan, and Hunan. Its stems and seeds are rich in lignans and triterpenoids, which can alleviated gastroenteric disorders and rheumatoid arthritis (Li et al. 2012). *K. coccinea* fruit is popular with people for its pleasing taste and health-beneficial effect. Previous studies have focused mainly on its medical properties, such as its anti-HIV (Pu et al. 2008), anti-lipid peroxidative (Gao et al. 2008), cytotoxic and anti-hepatitis (Zhao et al. 2014). However, only a few studies have focused on its phylogenetic evolution. We selected ‘black tiger 2’ with rose scent, which showed high resistance to drought, indicating that it has great potential as an excellent rootstock to resist drought. Here, The chloroplast (cp) genome of ‘black tiger 2’ was assembled, and it could facilitate study of the phylogenetic relationships between ‘black tiger 2’ and other plant species in the future.

Fresh ‘black tiger 2’ leaves were collected from Fruit Research Institute, Fujian Academy of Agricultural Sciences in Fuzhou (Fujian, China, 119°19′57″E, 26°7′47″N), and were deposited Fujian Agriculture and Forestry University (No.FAFUYSJ01). Total genomic DNA was extracted using the modified CTAB method (Jinlu et al. 2013). We then used the extracted genomic DNA to build an Illumina pair-end library. The library was sequenced using a HiSeq (Illumina, San Diego, CA, USA) at Beijing Genomics Institute (BGI, Shenzhen, China) and yielded approximately 3.66 GB of raw data. The paired-end raw data was filtered using the FastQC software (Andrews 2014). High-quality clean reads of around 3.64 G were used to assemble the cp genome using SPAdes v 3.9.0 (http://bioinf.spbau.ru/spades) (Bankevich et al. 2012) and the cp genome annotation was used with the online program GeSeq (Tillich et al. 2017). The annotated chloroplast (cp) genome of ‘black tiger 2’ has been deposited in Genbank under accession number MN480469.

The whole size of ‘black tiger 2’ chloroplast genome is 145,608 bp with overall GC content 38.6%, which contains a large single-copy region (LSC) of 94,457 bp, a small single-copy region (SSC) of 18,047 bp, and a pair of inverted repeat regions (IRA and IRB) of 16,552 bp. A total of 126 unique genes were predicted in the chloroplast genome, including 83 protein-coding genes, 35 transfer RNA (tRNA) genes, and 7 ribosomal RNA (rRNA) genes. In the IR regions, 4 rRNA species genes (rrn4.5, rrn5, rrn16, and rrn23) were found duplicated in each. Among all unique genes, 16 genes (trnK-UUU, rps16, trnG-UCC, atpF, rpoC1, trnL-UAA, trnV-UAC, petB, petD, rpl16, rpl2, ndhB, trnI-GAU, trnA-UGC, ndhA) contained one intron, whereas 2 genes (clpP, ycf3) contained two introns. The complete cp genome sequences of 22 species downloaded from the GenBank database were used to construct phylogenic maximum-likelihood trees using RaxML software v 8.2.9, of which the bootstrap values were calculated using 1000 replicates (Stamatakis 2014). Phylogenetic analysis indicated ‘black tiger 2’ was sister to *Schisandra chinensis*, within Austrobaileyales (Figure 1), which is similar to findings reported in a previous study (Li and Zheng 2018).

## Figures and Tables

**Figure 1.**
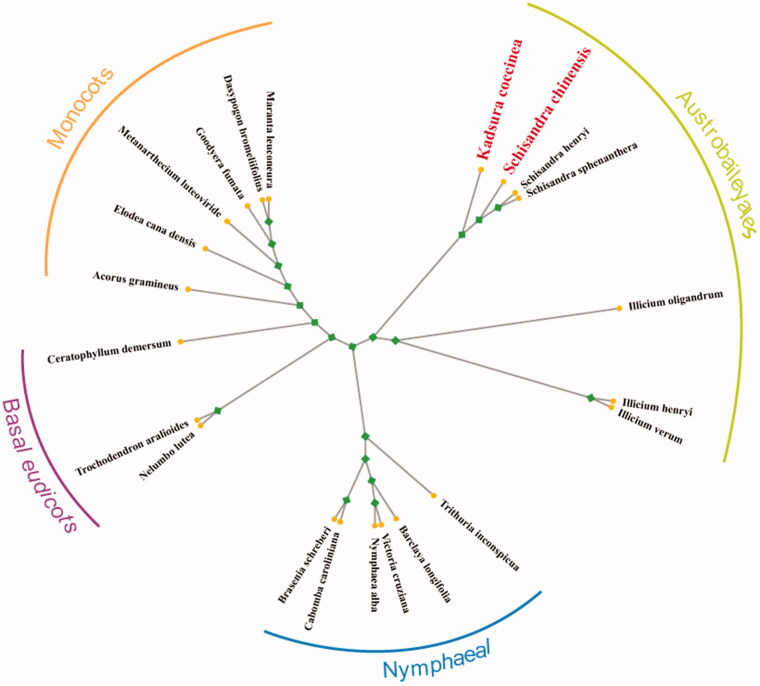
The maximum likelihood (ML) phylogenetic tree was constructed based on of the complete plants chloroplast genomes data of 22 species. The analyzed species and corresponding GenBank accession numbers are as follows: *Kadsura coccinea* (MN480469), *Schisandra chinensis* (NC 034908), *Schisandra sphenanthera* (NC_037145.1), *Schisandra henryi* (MH394370.1), *Illicium verum* (NC 034689), *Illicium oligandrum* (NC 009600), *Illicium henryi* (NC 034699), *Brasenia schreberi* (NC 031343), *Cabomba caroliniana* (NC 031505), *Trithuria inconspicua* (NC 020372), *Barclaya longifolia* (NC 035633), *Nymphaea alba* (NC 006050), *Victoria cruziana* (NC 035632), *Maranta leuconeura* (KF601571), *Dasypogon bromeliifolius* (JX088665), Goodyera fumata (KJ501999), *Metanarthecium luteoviride* (KT895904), *Acorus gramineus* (KP099646), *Elodea canadensis* (JQ310743), *Trochodendron aralioides* (KC608753), *Nelumbo lutea* (FJ754269) and *Ceratophyllum demersum* (AM712908).
